# A Carbapenem-Resistant *Pseudomonas aeruginosa* Isolate Harboring Two Copies of *bla*_IMP-34_ Encoding a Metallo-β-Lactamase

**DOI:** 10.1371/journal.pone.0149385

**Published:** 2016-04-07

**Authors:** Tatsuya Tada, Tohru Miyoshi-Akiyama, Kayo Shimada, Akino Shiroma, Kazuma Nakano, Kuniko Teruya, Kazuhito Satou, Takashi Hirano, Masahiro Shimojima, Teruo Kirikae

**Affiliations:** 1 Department of Infectious Diseases, Research Institute, National Center for Global Health and Medicine, 1-21-1 Toyama, Shinjuku, Tokyo, Japan; 2 Pathogenic Microbe Laboratory, Research Institute, National Center for Global Health and Medicine, 1-21-1 Toyama, Shinjuku, Tokyo, Japan; 3 Research and Development Division, Okinawa Institute of Advanced Sciences, Uruma, Okinawa, Japan; 4 Department of Accuracy Management, BML Inc., Kawagoe, Saitama, Japan; The University of Sydney, AUSTRALIA

## Abstract

A carbapenem-resistant strain of *Pseudomonas aeruginosa*, NCGM1984, was isolated in 2012 from a hospitalized patient in Japan. Immunochromatographic assay showed that the isolate was positive for IMP-type metallo-β-lactamase. Complete genome sequencing revealed that NCGM1984 harbored two copies of *bla*_IMP-34_, located at different sites on the chromosome. Each *bla*_IMP-34_ was present in the same structures of the class 1 integrons, *tnpA*(ISPa7)-*intI1*-*qacG*-*bla*_IMP-34_-*aac(6')-Ib*-*qacEdelta1*-*sul1*-*orf5*-*tniBdelta*-*tniA*. The isolate belonged to multilocus sequence typing ST235, one of the international high-risk clones. IMP-34, with an amino acid substitution (Glu126Gly) compared with IMP-1, hydrolyzed all β-lactamases tested except aztreonam, and its catalytic activities were similar to IMP-1. This is the first report of a clinical isolate of an IMP-34-producing *P*. *aeruginosa* harboring two copies of *bla*_IMP-34_ on its chromosome.

## Introduction

Metallo-β-lactamases (MBLs) produced by gram-negative bacteria confer resistance to all β-lactams, except monobactams, and are characterized by their efficient hydrolysis of carbapenems [1]. The most prevalent types of MBL are IMP-, NDM-, and VIM-type enzymes [1–3]. To date, 53 IMP-type MBLs have been registered (http://www.lahey.org/Studies/other.asp#table1). Of these, IMP-34 [4], IMP-41 [5], IMP-42 [5], IMP-43 [6], and IMP-44 [6] were recently identified in Japan.

The first gram-negative pathogen producing IMP-34 was a clinical strain of *Klebsiella oxytoca* showing intermediate resistance to imipenem, first isolated in 2013 in Japan [4]. The IMP-34 producer showed slightly decreased resistance to imipenem [4]. In this bacterium, *bla*_IMP-34_ was located on an 87343-bp plasmid, pKOI-34 (GenBank accession no. AB715422). There have been no previous reports regarding IMP-34-producing bacteria. At the amino acid sequence level, IMP-34 was found to have an amino acid substitution (Glu126Gly) compared with IMP-1, an amino acid substitution (Gly262Ser) compared with IMP-3, and two amino acid substitutions (Glu126Gly and Gly262Ser) compared with IMP-6 [4]. At the nucleotide sequence level, *bla*_IMP-1_ has two nucleotide sequence polymorphisms with four silent mutations at positions 189, 273, 496, and 702 (GenBank accession no. D50438 and AY250709). *bla*_IMP-34_ has two more nucleotide substitutions at positions 190 and 314. *bla*_IMP-3_ has an additional nucleotide substitution at position 640. Comparison of nucleotide sequence polymorphisms in these IMPs suggested that IMP-34 was evolutionarily close to IMP-1 [4].

Here, we describe a clinical isolate of carbapenem-resistant *P*. *aeruginosa* producing IMP-34, the complete genome sequence of the isolate, and the enzymatic properties of IMP-34.

## Materials and Methods

### Bacterial strains and drug susceptibility tests

*P*. *aeruginosa* NCGM1984 was obtained in 2012 from a urine sample of a patient hospitalized in Hyogo prefecture, Japan. *Escherichia coli* DH5α (Takara Bio, Shiga, Japan) and *E*. *coli* BL21-CodonPlus (DE3)-RIP (Agilent Technologies, Santa Clara, CA) were used as hosts for recombinant plasmids and for expression of *bla*_IMP-1_ and *bla*_IMP-34_.

Minimum inhibitory concentrations (MICs) were determined using the microdilution method, according to the guidelines of the Clinical Laboratory Standards Institute (CLSI) [7], i.e. the MIC breakpoints were for amikacin, ≤16 μg/mL for susceptibility (S) and ≥64 μg/mL for resistance (R); aztreonam, cefepime and ceftazidime, ≤8 μg/mL for S and ≥32 μg/mL for R; ciprofloxacin ≤1 μg/mL for S and ≥4 μg/mL for R; colistin, doripenem, imipenem, levofloxacin and meropenem, ≤2 μg/mL for S and ≥8 μg/mL for R; gentamicin and tobramycin, ≤4 μg/mL for S and ≥16 μg/mL for R; penicillin, ≤16 μg/mL for S and ≥128 μg/mL for R. The breakpoints were not determined by CLSI for ampicillin, ampicillin-sulbactam, arbekacin, cefotaxime, cefoxitin, cefozopran, cefpirome, ceftriaxone, cefuroxime, cphradine fosfomycin, moxalactam and tigecycline.

### Detection and sequencing of IMP-type MBLs, AAC(6')-Iae, and AAC(6')-Ib

Multidrug-resistant *P*. *aeruginosa* isolates producing IMP-type MBLs, AAC(6′)-Iae, and AAC(6')-Ib were screened using immunochromatographic assay kits for detection of IMP-type MBLs [8] (Mizuho Medy Co., Saga, Japan), AAC(6′)-Iae [9], and AAC(6′)-Ib [10] (Mizuho Medy Co.), respectively. We routinely perform assays to detect these producers, because the majority of multidrug-resistant *P*. *aeruginosa* clinical isolates in Japan produce these antibiotic resistance factors, i.e., the rates of IMP producers and AAC(6′)-Iae/AAC(6′)-Ib producers among multidrug-resistant *P*. *aeruginosa* isolates were 76.7% and 77.8%, respectively, in 2012 in Japan [11]. The *bla*_IMPs_ genes were amplified using PCR primers as described [8]. All PCR products were sequenced using an ABI PRISM 3130 sequencer (Applied Biosystems, Foster City, CA).

### Complete genome sequencing

The entire genome of *P*. *aeruginosa* NCGM1984 was extracted with cetyl-trimethylammonium bromide (CTAB), sequenced using PacBio RSII (Pacific Biosciences, Menlo Park, CA), and assembled using Minimus 2 to determine the complete genome sequence. Multilocus sequence typing (MLST) was determined according to the *P*. *aeruginosa* MLST Database website (http://pubmlst.org/paeruginosa/). RAST automated annotation servers (http://rast.nmpdr.org/) were used for primary coding sequence (CDS) extraction and initial functional assignment. CDS annotations were confirmed using *In Silico* Molecular Cloning software (In Silico Biology, Inc., Kanagawa, Japan), which assists in annotation with comparison to sequences registered in GenBank.

### Comparative genome analysis

The genome sequences of *P*. *aeruginosa* PAO1 and NCGM2.S1 strains (accession no. AE004091 and AP012280, respectively) were used for comparative genome analysis with the sequence of NCGM1984. Genomic islands harboring a class 1 integron(s) were detected by comparison with the sequence of PAO1 strain. The sequence of NCGM2.S1 was used as the reference strain belonging to ST235.

### Cloning of *bla*_IMPs_

*bla*_IMP-1_ and *bla*_IMP-34_ were cloned into *E*. *coli* DH5α as described [6]. The ORFs of *bla*_IMP-1_ and *bla*_IMP-34_ were amplified by PCR using the primers EcoRI-IMP-1/34-F(5′-gggGAATTCatgagcaagttatctgtattc-3′; uppercase letters indicate an *Eco*RI digestion site) and PstI-IMP-1/34-R (5′-aaaCTGCAGttagttgcttggttttgatgg-3′; uppercase letters indicate a *Pst*I digestion site). The PCR products were digested with *Eco*RI and *Pst*I and ligated into pHSG398 (Takara Bio, Shiga, Japan). The plasmids were used to transform DH5α, and transformants were selected on LB agar containing 30 μg/mL of chloramphenicol, and their susceptibilities to various β-lactams were assayed. *P*. *aeruginosa* NCGM2.S1 harboring *bla*_IMP-1_ was used as a reference strain [12].

### Enzymatic activities of recombinant IMPs

Recombinant IMP-1 and IMP-34 were purified as described [6]. *bla*_IMP-1_ and *bla*_IMP-34_ were amplified using the primers BamHI-IMP-1/34-F (5′-atGGATCCgaaaacctgtatttccaaggcgcagagtctttgccagattt-3′; uppercase letters indicate a *Bam*HI digestion site) and XhoI-IMP-1/34-R (5′-atcCTCGAGttagttgcttggttttgatgg-3′; uppercase letters indicate an *Xho*I digestion site). These PCR products were digested with *Bam*HI and *Xho*I and ligated into pET28a (Novagen, Inc., Madison, WI). The plasmids were used to transform *E*. *coli* BL21-CodonPlus (DE3)-RIP (Agilent Technologies, Santa Clara, CA), and transformants were selected on LB agar containing 20 μg/mL of kanamycin. The bacterial cells were lysed by sonication and the recombinant IMP proteins were purified from the soluble fraction on Ni-NTA agarose according to the manufacturer’s instructions (Qiagen, Tokyo, Japan). His-tagged proteins were digested with TurboTEV protease (Accelagen, San Diego, CA), and both the His-tag and the protease were removed on Ni-NTA agarose. SDS-PAGE analysis showed that each target protein was obtained with >90% purity. During the purification procedures, the presence of β-lactamase activities was monitored with 100 μM nitrocefin (Oxoid Ltd., Basingstoke, UK). Kinetic analysis was performed in 50 mM Tris-HCl buffer (pH 7.4) containing 5 μM Zn(NH_3_)_2_ at 37°C using a UV-visible spectrophotometer (V-530; Jasco, Tokyo, Japan). The *Km*, *k*cat, and *k*cat/*Km* ratio of each enzyme were determined by analyzing β-lactam hydrolysis under initial-rate conditions using Lineweaver–Burke plots [13–15].

### Nucleotide sequence accession numbers

The complete genome sequence of NCGM1984 has been deposited in GenBank under the accession number AP014646.

### Ethical statements

The study protocol was carefully reviewed and approved by the ethics committee of the National Center for Global Health and Medicine (No. 1268). Individual informed consent was waived by the ethics committee listed above because this study used currently existing samples collected during the course of routine medical care and did not pose any additional risks to the patients. Patient information was anonymized and de-identified prior to analysis. The study protocol was reviewed and approved by the Biosafety Committee, National Center for Global Health and Medicine (approval numbers: 26-D-088 and 26-D-089).

## Results

### Antibiotic susceptibility of *P*. *aeruginosa* NCGM1984

*P*. *aeruginosa* NCGM1984 was resistant to all β-lactams tested (Table 1). In particular, the isolate was extremely resistant to imipenem and meropenem, with MICs of 512 μg/mL and >1,024 μg/mL (the breakpoints for both antibiotics: ≥8 μg/mL for R), respectively (Table 1). The MICs of other antibiotics were as follows: amikacin, 16 μg/mL (≥64 μg/mL for R); arbekacin, 128 μg/mL (no criteria for the breakpoint); gentamicin, 16 μg/mL (≥16 μg/mL for R); tobramycin, 128 μg/mL (≥16 μg/mL for R); ciprofloxacin, 64 μg/mL (≥4 μg/mL for R); colistin, 0.5 μg/mL (≥8 μg/mL for R); fosfomycin, >1,024 μg/mL (no criteria); levofloxacin, 128 μg/mL (≥8 μg/mL for R); and tigecycline, 16 μg/mL (no criteria).

**Table 1:** MICs of β-lactams for *P*. *aeruginosa* NCGM1984 and *E*. *coli* transformants with *bla*_IMP-1_ and *bla*_IMP-34_.

Antibiotic(s)*^a^*	MIC (μg/ml)			
	*P*. *aeruginosa* NCGM1984	*E*. *coli* DH5α (pHSG398/IMP-1)	*E*. *coli* DH5α (pHSG398/IMP-34)	*E*. *coli* DH5α (pHSG398)
Ampicillin	> 1,024	32	32	2
Ampicillin-sulbactam*^b^*	1,024 (683/341)	16 (11/5)	16 (11/5)	1 (0.7/0.3)
Aztreonam	64	≤ 0.25	≤ 0.25	≤ 0.25
Cefepime	> 1,024	2	2	2
Cefotaxime	> 1,024	16	16	≤ 0.25
Cefoxitin	> 1,024	512	512	2
Cefozopran	> 1,024	4	4	≤ 0.25
Cefpirome	512	0.25	0.5	≤ 0.25
Ceftazidime	> 1,024	512	512	≤ 0.25
Ceftriaxone	> 1,024	64	64	≤ 0.25
Cefuroxime	> 1,024	256	128	4
Cephradine	> 1,024	256	256	16
Doripenem	> 1,024	0.25	≤ 0.25	≤ 0.25
Imipenem	512	0.5	0.25	≤ 0.25
Meropenem	> 1,024	0.5	0.25	≤ 0.25
Moxalactam	> 1,024	64	64	≤ 0.25
Penicillin G	> 1,024	128	128	32

^*a*^The breakpoints were for aztreonam, cefepime and ceftazidime, ≥32 μg/ml for R; dorpiemen, imipenem and meropenem, ≥8 μg/ml for R; and penicillin G, ≥128 μg/ml for R. The breakpoints were not determined for other antibiotics listed in Table 1.

^*b*^The ratio of the ampicillin to sulbactam was 2:1. The MICs are shown as concentrations of compounds combined with ampicillin and sulbactam (concentrations of ampicillin/concentrations of sulbactam).

### Identification of *bla* IMP-34 in *P*. *aeruginosa* NCGM1984

Immunochromatographic assays showed that *P*. *aeruginosa* NCGM1984 was positive for IMPs and AAC(6')-Ib, but negative for AAC(6')-Iae. The isolate harbored *bla*_IMP-34_ and *aac(6')-Ib*. IMP-34, which had an amino acid sequence substitution (Glu126Gly) compared with IMP-1, belongs to the IMP-1-like group.

### Complete genome analysis of *P*. *aeruginosa* NCGM1984

The complete genome of *P*. *aeruginosa* NCGM1984 was obtained with 533-fold coverage. This genome consisted of a single circular chromosome, 6,850,954 bp in size, with an average GC content of 65.96%. The chromosome contained a total of 6,282 CDS, 66 tRNA genes, and 1 tmRNA for all amino acids. NCGM1984 had no plasmids. The chromosome contained three integrons, two of which were located close to each other in an area of lower GC content, whereas the other was not. The MLST of NCGM1984 was ST235.

NCGM1984 had a gene associated with β-lactam resistance, *bla*_IMP-34_, and three genes associated with aminoglycoside resistance, *aac(6')-Ib*, *aacA7*, and *aadA6*. In addition, the isolate had two point mutations in the quinolone resistance-determining regions of *gyrA* and *parC*, with amino acid substitutions of Thr83Ile in GyrA and Ser87Leu in ParC, which are associated with quinolone resistance [16,17], and had *fosA*, which is associated with fosfomycin resistance. NCGM1984 also had intrinsic β-lactamase encoding genes, *bla*_OXA-50_ and *bla*_PDC-20_, which are not thought to be associated with β-lactam resistance [18].

**Fig 1:**
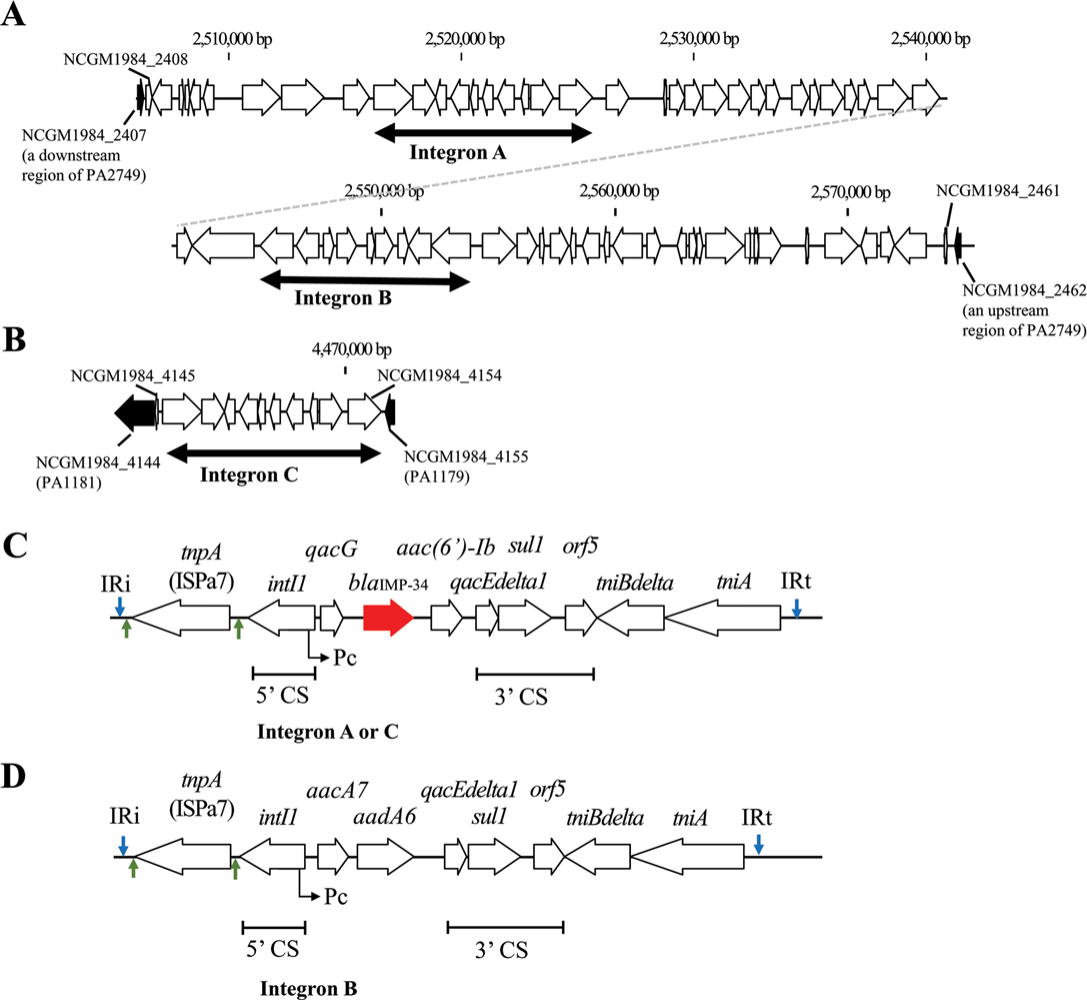
Genomic islands containing class 1 integrons in *Pseudomonas aeruginosa* NCGM1984. (A) Genomic island containing integrons A and B. The genomic island was inserted between NCGM1984_2402 (PA2752 in the complete genome of *P*. *aeruginosa* PAO1: accession no.AE004091) and NCGM1984_2462 (PA2749). (B) Genomic island containing integron C. The genomic island was inserted between NCGM1984_4147 (PA1181) and NCGM1984_4144 (PA1179). (C) The genomic structure of integrons A and C. The structure of integron A was identical to that of integron C. These integrons contained *bla*_IMP-34_. The red arrow indicates *bla*_IMP-34_. Blue arrows indicate IRi and IRt. Green arrows indicate direct repeats. (D) Genomic structure of integron B. Blue arrows indicate IRi and IRt. Green arrows indicate direct repeats.

*bla*_IMP-34_ was detected on the chromosome of NCGM1984. Unexpectedly, NCGM1984 harbored two copies of *bla*_IMP-34_, located at different sites on the chromosome (nt 2,521,570 – 2,522,310 and nt 4,467,459 – 4,468,199). A copy of *bla*_IMP-34_ was located on a large genomic island of 65,600 bp between PA1984_2407 and PA1984_2462 (Fig 1A). The large genomic island contained two class 1 integrons, integrons A and B (Fig 1A). A copy of *bla*_IMP-34_ was located on integron A. Another copy of *bla*_IMP-34_ was located on a small genomic island of 9,408 bp between NCGM1984_4145 and NCGM1984_4155 (Fig 1B). This small genomic island was another class 1 integron, integron C, and contained no other CDS (Fig 1B). The small genetic island was located between putative sensor protein encoding gene, *yegE*, and two-component response regulator encoding gene, *phoP*. The genetic structures of integrons A and C containing *bla*_IMP-34_ were identical to each other (Fig 1C). All three integrons had a pair of inverted repeats, IRi and IRt (Fig 1C).

Integron A (from IRi to IRt) was located on nt 2,516,107 – 2,525,888 (9,782 bp) and integron C (from IRi to IRt) was located on nt 4,461,996 – 4,471,777 (9,782 bp). The integron A was not flanked by 5 bp dupulication, whereas the integron C had made by a duplication (CAGGT in nt 4,461,991–4,461,995 and 4,471,778–4471782). The class 1 integrons A and C carried the *qacG-blaIMP-aac(6')-Ib* cassettes. In integrons A and C, the 5'-CS was interrupted by ISPa7 (Fig 1C). The class 1 integron B (from IRi to IRt) was located on nt 2,544,427 – 2,553,926 (9,500 bp), and carried the *aacA7*-*aadA6* cassette. The integron B was not flanked by 5 bp duplication.

Integrons A and B had a Pc promoter with TGGACA (–35 sequence) and TAAACT hexamers (–10 sequence) separated by a space of 17 bp within *intI1*, respectively; whereas integron C had another Pc promoter with TGGACA (–35 sequence) and TAAGCT hexamers (–10 sequence) separated by a space of the same size (17 bp) [19].

### Drug susceptibility of *E*. *coli* DH5α expressing IMP-34 and enzymatic activities

The drug susceptibility profile of *E*. *coli* expressing IMP-34 was similar to that of *E*. *coli* expressing IMP-1, although the former showed slightly lower MICs for doripenem, imipenem, and meropenem compared with the latter (Table 1).

Recombinant IMP-34, as well as IMP-1, hydrolyzed all β-lactams tested except aztreonam (Table 2). The *k*_cat_*/K*_m_ ratios of IMP-34 were similar to those of IMP-1 against all β-lactams (Table 2).

**Table 2:** Kinetic parameters of the β-lactamases IMP-1 and IMP-34 with various substrates.

Substrate	*Km* (μM)*^a^*		*kcat* (s^-1^)*^a^*		*kcat*/*Km* (μM^-1^・s^-1^)*^a^*	
	IMP-1	IMP-34	IMP-1	IMP-34	IMP-1	IMP-34
Penicillin G	685 ± 99	486 ± 71	94 ± 12	55 ± 7	0.14	0.11
Ampicillin	341 ± 17	360 ± 58	16 ± 1	13 ± 1	0.048	0.037
Cephradine	73 ± 2	57 ± 6	21 ± 1	15 ± 1	0.29	0.26
Cefoxitin	34 ± 5	31 ± 1	2.7 ± 0.1	2.0 ± 0.1	0.080	0.066
Cefotaxime	13 ± 2	12 ± 1	2.8 ± 0.1	2.0 ± 0.1	0.22	0.18
Ceftazidime	26 ± 1	22 ± 5	0.68 ± 0.01	0.41 ± 0.03	0.026	0.020
Cefepime	22 ± 8	29 ± 3	1.4 ± 0.2	2.4 ± 0.1	0.068	0.084
Aztreonam	NH*^b^*	NH*^b^*	NH*^b^*	NH*^b^*	NH*^b^*	NH*^b^*
Dripenem	39 ± 8	39 ± 12	4.8 ± 0.1	3.8 ± 0.2	0.13	0.100
Imipenem	58 ± 14	58 ± 5	6.9 ± 0.5	4.9 ± 0.4	0.12	0.084
Meropenem	37 ± 12	46 ± 4	2.3 ± 0.2	1.99 ± 0.05	0.066	0.043

^*a*^The *K*m and *k*cat values represent the means± standard deviations of three independent experiments.

^*b*^NH: no hydrolysis was detected at substrate concentrations of up to 1 mM and enzyme concentrations of up to 700 nM.

## Discussion

The Glu126Gly substitution in IMP-34 slightly affected the catalytic efficiency of the enzyme for β-lactam hydrolysis compared with IMP-1 (Table 2). The amino acid residue at position 126 is located in the same helix as position 120, which is a Zn^2+^ binding site [20]. There have been no previous reports regarding the role of the amino acid residue at position 126 of IMPs.

IMPs in multidrug-resistant *P*. *aeruginosa* clinical isolates in Japan seem to have increased efficiency of catalytic activities against carbapenems due to the acquisition of various amino acid substitutions. For example, multidrug-resistant *P*. *aeruginosa* producing IMP-43 and IMP-44 showed greater catalytic activities against carbapenems than IMP-7 and IMP-11, respectively [6]. IMP-43 belonging to the IMP-7-like group has an amino acid substitution (Val67Phe) compared with IMP-7, and IMP-44 belonging to the IMP-11-like group has two substitutions (Val67Phe and Phe87Ser) compared with IMP-11. IMP-43 showed more efficient catalytic activities against doripenem, imipenem, and meropenem than IMP-7, while IMP-44 had more efficient catalytic activities against all carbapenems compared to IMP-11 [6].

The activity of the Pc promoter of integron A will be stronger than that of integron C [21–23]. There are four versions of Pc, designated as “weak,” “strong,” “hybrid 1,” and “hybrid 2,” which show differences in the –35 and /or –10 sequences, separated by 17 bases [21–23]. The Pc promoter of integron A was “hybrid 1” type, and that of integron C was “weak” type. It was reported that there are eight variants of the Pc promoter, which vary in their promoter activities [23]. The –35/–10 sequences of PcS (strong type) were TTGACA/TAAACT, whereas those of PcW (weak type) were TGGACA/TAAGCT. The promoter activity of the hybrid type 1 was 4.5-fold weaker than that of the strong type and 5.6-fold stronger than that of the weak type [22]. The Pc promoter of integron A and B was a hybrid type consisting of a weak type –35 sequence combined with a strong type–10 sequence (TGGACA/TAAACT). Two copies of *bla*_IMP-34_ in NCGM1984 will contribute to extremely high MICs of carbapenems, although these copies will express at different levels because of different Pc promoters of the integrons and the Pc promoter of integron A together with that of integron C may be the main determinant of the level of carbapenem resistance.

Multidrug-resistant *P*. *aeruginosa* belonging to ST235, including NCGM1984, is recognized as one of the international high-risk clones that spread in medical settings worldwide, which has acquired nearly 100 resistance elements, including resistance to 39 different β-lactamases [24]. Multidrug-resistant *P*. *aeruginosa* belonging to ST235 spread in Japan and often carried an In113-like integron, harboring *bla*_IMP-1_, *aac(6')-Iae*, and *aadA1a* [25]. These horizontally acquired antibiotic resistance elements exist in various integrons in the multidrug-resistant isolates belonging to ST235 [24] as well as NCGM1984 (Fig 1C and 1D).

The large genomic island with two class 1 integrons found in NCGM1984 had a quite unique structure (Fig 1A) and seemed to originate from three different genomic islands. The large genomic island in NCGM1984 had only 56% similarity with *P*. *aeruginosa* C79 genomic island (accession no. JF826498) at the most; nevertheless, its partial structures divided by the two integrons had higher similarity with other *P*. *aeruginosa* genomes as follows: the upstream region of integron A (NCGM1984_2402 to _2410) had 99% identity with *P*. *aeruginosa* H47921 isolated in the United States; the region between integrons A and B (NCGM1984_2420 to _2436) had 100% similarity with *P*. *aeruginosa* VR-143/97 genomic island isolated in Italy (accession no. LK054503); the downstream region of integron B (NCGM1984_2446 to _2461) had 99% similarity with *P aeruginosa* C79 isolated in Australia (accession no. JF826498).

In addition to the acquisition of amino acid substitutions in IMPs, increases in the *bla*_IMP_ copy number may represent an alternative pathway by which *P*. *aeruginosa* can acquire carbapenem resistance. That is, the extreme resistance of NCGM1984 against carbapenems may be associated with the presence of two copies of *bla*_IMP-34_. Amplification of genes conferring bacterial resistance to antibiotics in bacteria may be associated with a gene dosage effect [26]. For example, a clinical isolate of *P*. *aeruginosa* carrying two copies of *bla*_NDM-1_ on the chromosome [27] was found to be extremely resistant to imipenem and meropenem (MIC > 32 μg/mL) [28].
